# A Novel sEMG-Based Gait Phase-Kinematics-Coupled Predictor and Its Interaction With Exoskeletons

**DOI:** 10.3389/fnbot.2021.704226

**Published:** 2021-08-10

**Authors:** Baichun Wei, Zhen Ding, Chunzhi Yi, Hao Guo, Zhipeng Wang, Jianfei Zhu, Feng Jiang

**Affiliations:** ^1^School of Computer Science and Technology, Harbin Institute of Technology, Harbin, China; ^2^Pengcheng Laboratory, Shenzhen, China; ^3^School of Mechatronics Engineering, Harbin Institute of Technology, Harbin, China

**Keywords:** electromyography, motion decoding algorithm, kinematics prediction, gait recognition, long short-term memory

## Abstract

The interaction between human and exoskeletons increasingly relies on the precise decoding of human motion. One main issue of the current motion decoding algorithms is that seldom algorithms provide both discrete motion patterns (e.g., gait phases) and continuous motion parameters (e.g., kinematics). In this paper, we propose a novel algorithm that uses the surface electromyography (sEMG) signals that are generated prior to their corresponding motions to perform both gait phase recognition and lower-limb kinematics prediction. Particularly, we first propose an end-to-end architecture that uses the gait phase and EMG signals as the priori of the kinematics predictor. In so doing, the prediction of kinematics can be enhanced by the ahead-of-motion property of sEMG and quasi-periodicity of gait phases. Second, we propose to select the optimal muscle set and reduce the number of sensors according to the muscle effects in a gait cycle. Finally, we experimentally investigate how the assistance of exoskeletons can affect the motion intent predictor, and we propose a novel paradigm to make the predictor adapt to the change of data distribution caused by the exoskeleton assistance. The experiments on 10 subjects demonstrate the effectiveness of our algorithm and reveal the interaction between assistance and the kinematics predictor. This study would aid the design of exoskeleton-oriented motion-decoding and human–machine interaction methods.

## Introduction

For the past few decades, with the development of human–machine interaction and human motion-decoding methods, an advanced technology was developed to bridge the gap between the human and robots (Bonato, 2010). This robotic technology, known as the wearable robot, directly interacts with the human body to enhance the mobility of healthy people (exoskeletons), to treat muscles or skeletal parts which are injured or after the operation (orthosis), or to replace the missing limbs of disabled people (prostheses) (Viteckova et al., 2013; Chadwell et al., 2020).

As an important branch of wearable robots, the lower-limb exoskeletons run in parallel to the human lower-limbs, with representative applications to daily assistance, medical rehabilitation, and other areas (Kazerooni, 2008; Sankai, 2010; Awad et al., 2017). In recent years, with the development of human-machine interaction technology and advanced wearable sensors, the exoskeletons have been able to decode the human motions based on physiological or kinematic signals, meanwhile autonomously and promptly assist the user's locomotion at the critical timing, which has enhanced the initiative and intelligence of the system (Yan et al., 2015).

Surface electromyography (sEMG), one of the commonly used neural signals for motion-decoding, integrates the spatial and temporal information of the muscles (Joshi et al., 2013). The amplitude of sEMG is highly related to the level of muscle activation, owing to which sEMG is widely used in control strategies of exoskeletons (Yang et al., 2008; Fan and Yin, 2009). The traditional and practical control strategy for exoskeletons and prostheses is known as the ‘direct myoelectric control’ approach. The strategy collects the sEMG signals to control the motors of the mechanical joints (Williams, 1990). Although this control strategy has achieved considerable reliability, it becomes non-intuitive when the number of mechanical joints increases. The user training process also tends to be quite time-consuming and cumbersome (Resnik et al., 2018).

**Figure 1:**
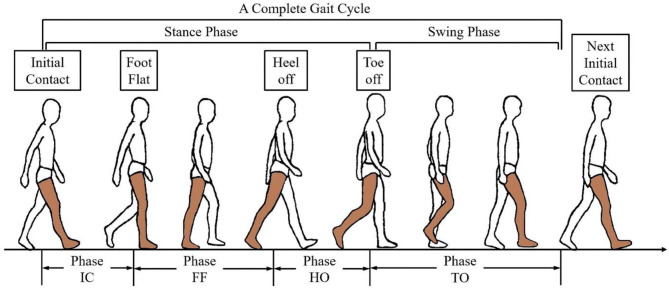
The gait phase definition in a gait cycle.

As a potential solution to the problem, sEMG-based pattern recognition methods have been developed for motion-decoding and myoelectric control, which seeks the synergistic relationship between muscles based on multichannel sEMG signals, and then matches it with the defined patterns (Scheme and Englehart, 2011). For lower-limb exoskeletons, the motion pattern that is necessary for achieving the mode switching of the control system is the gait phase, which may help to provide a more proper assistant force on human movement (Vu et al., 2018). One of the commonly used gait phase definitions for exoskeletons is shown in Figure 1, which segments the gait cycle based on several significant events, such as the initial contact or the toe off (Taborri et al., 2016).

As a general rule, the sEMG-based phase classification process includes extracting the temporal or spatial-temporal features from window-segmented sEMG signals, followed by a classifier to align the features to the pre-defined phases (Novak and Riener, 2015). Compared with the non-stationary raw sEMG signals, the feature-extraction process maximally separates the desired output classes, with an impressive performance in pattern recognition (Hudgins et al., 1993). However, the feature representation will lead to the increased dimension of data, which may increase the burden to the limited computing equipment of the exoskeleton. Dimension-reduction plays an important role in the related research, with representative methods such as principal component analysis (PCA) (Englehart et al., 2001), linear discriminant analysis (LDA) (Chu et al., 2007), and profile likelihood maximization (Naik et al., 2018). Although various methods were proposed to deal with the ‘curse of dimension’ problem in the feature space, few studies focused on the source data space, i.e., the selected muscles in the studies. Dealing with the muscle redundancy problem, i.e., removing the muscles that have less effect on phase recognition, will reduce not only the dimension of the input data but also the number of sensors.

Due to the motion continuity, the kinematics of the lower-limb joints is time-varying in a gait phase. In addition, the mode switching of the control system may diminish the continuity and smoothness of assistance during the transition of different phases (Kim et al., 2019). Thus, continuous decoding of lower-limb kinematics is beneficial to provide additional knowledge for more precise exoskeleton control. So far, extensive work has been done to estimate the joint kinematics, such as joint angles (Ngeo et al., 2014) of trajectories (Xia et al., 2018). However, when considering the limited computing power of the exoskeletons, the application of these methods may cause a time delay between the estimated kinematics and the actual occurrence of the motion event, which may reduce the effectiveness of the exoskeleton and even cause a potential injury to the subject (Tanghe et al., 2020). In order to compensate for this time delay, our previous work achieved the ahead-of-time prediction of kinematics (Yi et al., 2021). However, the study did not consider the simultaneous classification of the gait phases, which would be beneficial for kinematics prediction because of the common quasi-periodicity.

For exoskeletons, there exists another problem in applications of sEMG-based motion-decoding methods. According to Sylos-Labini et al. (2014), the assistive forces provided by an exoskeleton may result in a change of the muscle coordination manners (i.e., muscle synergies). Similar conclusions were also given by the related studies that investigated the effect of robotic gait assistance on the muscle function of the subjects (Moreno et al., 2013; Li et al., 2019). The altered muscle functions would cause an unknown distribution change of sEMG, therefore cause adverse effects on the sEMG-based motion-decoding methods. However, there is still a lack of investigation of how the exoskeleton affects the sEMG-based motion decoding methods, which matters a lot for the applications of the methods to exoskeletons.

In this study, we propose a novel motion-decoding method that combines the recognition of gait phases and the prediction of lower-limb joint angles. The main contributions of this paper are integrated as follows:

- We propose an sEMG and gait phase-based continuous lower-limb kinematics predictor, which leverages not only the ahead-of-motion property of sEMG but also the quasi-periodicity of gait phases to present the ahead-of-time joint kinematics prediction.
- We propose a muscle selection scheme in view of the effects of muscles on the classification of gait phases.
- We experimentally quantify how the assistance of an ankle exoskeleton affects the motion-decoding methods and propose a fine-tuning scheme to adapt to the performance degradation caused by exoskeleton assistance.

The structure of the paper is as follows. In Related Works section, the related works are briefly described. Materials and Methods section details the data acquisition process, the experimental design, and the structure of the proposed motion-decoding method. The evaluation metrics validating the effectiveness of our method are also described in this section. The experimental results are detailed in Results section and analyzed in Discussion section. The conclusion underlines the performance of the proposed method in Conclusion section.

## Related Works

### Phase Recognition and Dimension Reduction

The gait phase recognition is a non-trivial problem for exoskeleton and prosthesis, which is used to permit the control system to work with more initiative and precision (Ferris et al., 2007). Joshi et al. presented a method that combined the Bayesian information criterion and LDA to recognize eight phases based on four-channel EMG signals (Joshi et al., 2013). With an average accuracy of 76.12%, the recognized phases were applied in an exoskeleton orthosis. The study in (Ryu and Kim, 2014) implemented fractal analysis to analyze the change of vibroathrographic signals. Based on four-channel EMG signals, the support vector machine (SVM) classifier could recognize four phases with an average accuracy of 91%.

In recent years, deep learning has revolutionized the fields correlated with machine learning and pattern recognition (LeCun et al., 2015). Compared with other machine learning methods, deep learning is better at searching for the relations of the source data with the labels. In addition, the change of the gait phase is quasi-periodic, which means the temporal-contextual data is beneficial for phase recognition. Because of the reasons described above, we adopted the Long-Short Term Memory (LSTM) to design the phase classifier.

For exoskeleton systems, the motion-decoding algorithms usually run on an onboard microcomputer, which means the source data need to be carefully selected to avoid the control system hysteresis caused by high computational complexity. Moreover, the feature extraction process increases the dimension of the input data by multiples, which may add another layer of complexity. Thus, dimension reduction usually plays an important role in exoskeleton systems. The study in Chu et al. (2007) compared different feature projection methods, such as LDA and PCA, and evaluated through Sammon's stress and Fisher's index. A study by Naik et al. (2018) introduced a screen-plot-based statistical technique for feature reduction. With the implementation of the Fisher score, the method reduced the feature dimension from 28 to 13.

Although various dimension reduction methods have been proposed to avoid the model overfitting and reduce computational complexity, few studies have analyzed the selected muscles. In their works, the muscles were mostly determined by related works or experiences. In this study, we propose a muscle selection scheme that analyzes the effects of muscles on phase classification. Through this scheme, the redundant muscles will be discarded in order to both reduces the dimension of the data and the number of the sensors.

### Continuous Decoding of Joint Kinematics

Because most of the lower-limb exoskeletons are located at the joints, such as the knee or the ankle, it is beneficial to obtain the kinematic parameters of the joints, which provide more continuous and detailed knowledge for smooth control. See et al. solved the joint axis using the numerical optimization method, established the limb coordinate system, and calculated the lower limb joint angle based on the IMU signals (Seel et al., 2014). Ameri et al. proposed a real-time upper limb wrist joint trajectory decoding method based on support vector regression (SVR). They implemented this method for proportional control based on EMG signals (Ameri et al., 2014). In the study of Xia et al. (2018), a deep architecture-based model was proposed to estimate the limb trajectory, which combined the convolutional neural network (CNN) and recurrent neural networks (RNN). The results showed that the accuracy and robustness of the proposed method are much higher than those of SVR and CNN.

Although the above studies have shown considerable performance, the time delay in control hinders their application to exoskeleton systems. In order to deal with this problem and enhance the control of exoskeleton, the future joint kinematics are required. Kevin et al. proposed a probabilistic model to present the future prediction of the current kinematics and gait events, which leveraged the quasi-periodicity of lower-limb motions (Tanghe et al., 2020). The method presented a pioneer frame for kinematic prediction, and it can be enhanced by physiological knowledge. According to the previous studies, there exists a time delay between the onset of the sEMG and the occurrence of the movement (Hioki and Kawasaki, 2012). This phenomenon, known as the electromechanical delay (EMD), can be helpful for the ahead-of-time prediction of kinematics. Thus, we propose an LSTM-based lower-limb kinematic predictor, which leverages the quasi-periodicity of phase and EMD to present the ahead-of-time lower-limb joint angles.

### Effects of Exoskeletons on Muscle Functions

How the exoskeletons affect the muscle functions of the subjects have been investigated for many years (Steele et al., 2017). Prior studies have revealed that external forces that were provided by the exoskeletons would alter the activity-level and recruitment patterns of the muscle groups (Sylos-Labini et al., 2014; Li et al., 2019). The study (Sylos-Labini et al., 2014) recorded the sEMG activity of six healthy individuals during overground walking with a lower-limb exoskeleton. The result revealed that the activity of some muscles increased in the exoskeleton-assisted condition compared with the normal walking condition, while the other muscles did not change significantly. Pearson correlation coefficients were implemented as another metric to compare the sEMG waveforms in these two conditions, and a significant difference was found. In Steele et al. (2017), muscle synergy and muscle activity were implemented to evaluate the changes in muscle recruitment and coordination patterns. The result revealed that the subjects could selectively modulate the activity of individual muscles and were not constrained to synergistic patterns of muscle coordination.

The related studies designed complete experiments to investigate the effects of exoskeleton on muscle functions, and concluded that exoskeletons could alter the muscle recruitment patterns (Li et al., 2019). However, there is still a lack of research on the investigation of the exoskeleton effect on sEMG-based motion-decoding methods. Such effect is worthy of investigation since sEMG has obvious advantages in application to exoskeletons, such as the EMD and information of kinematics, dynamics, and personal identity. Thus, we experimentally quantified the effect of an ankle exoskeleton on the proposed motion-decoding model. Also, we implemented a fine-tuning scheme to allow the model to adapt to the change of data distribution caused by exoskeletons' assistance.

## Materials and Methods

### Data Acquisition and Experimental Protocol

This study was conducted under the approval of the Chinese Ethics Committee of Registering Clinical Trials, and all the subjects signed the consent form corresponding to the experiments, who could decide to stop the experiment at any time. The subjects include 10 healthy males with an average height of 178 ± 5 cm and an average weight of 77.6 ± 10 kg. The data collection was performed using the EMG acquisition equipment (Delsys Trigno, IM type and Avanti type), a designed foot pressure acquisition device, and an optical motion capture system (VICON). At the beginning of the data collecting process, the signals from various acquisition devices were synchronized by a trigger device.

**Figure 2:**
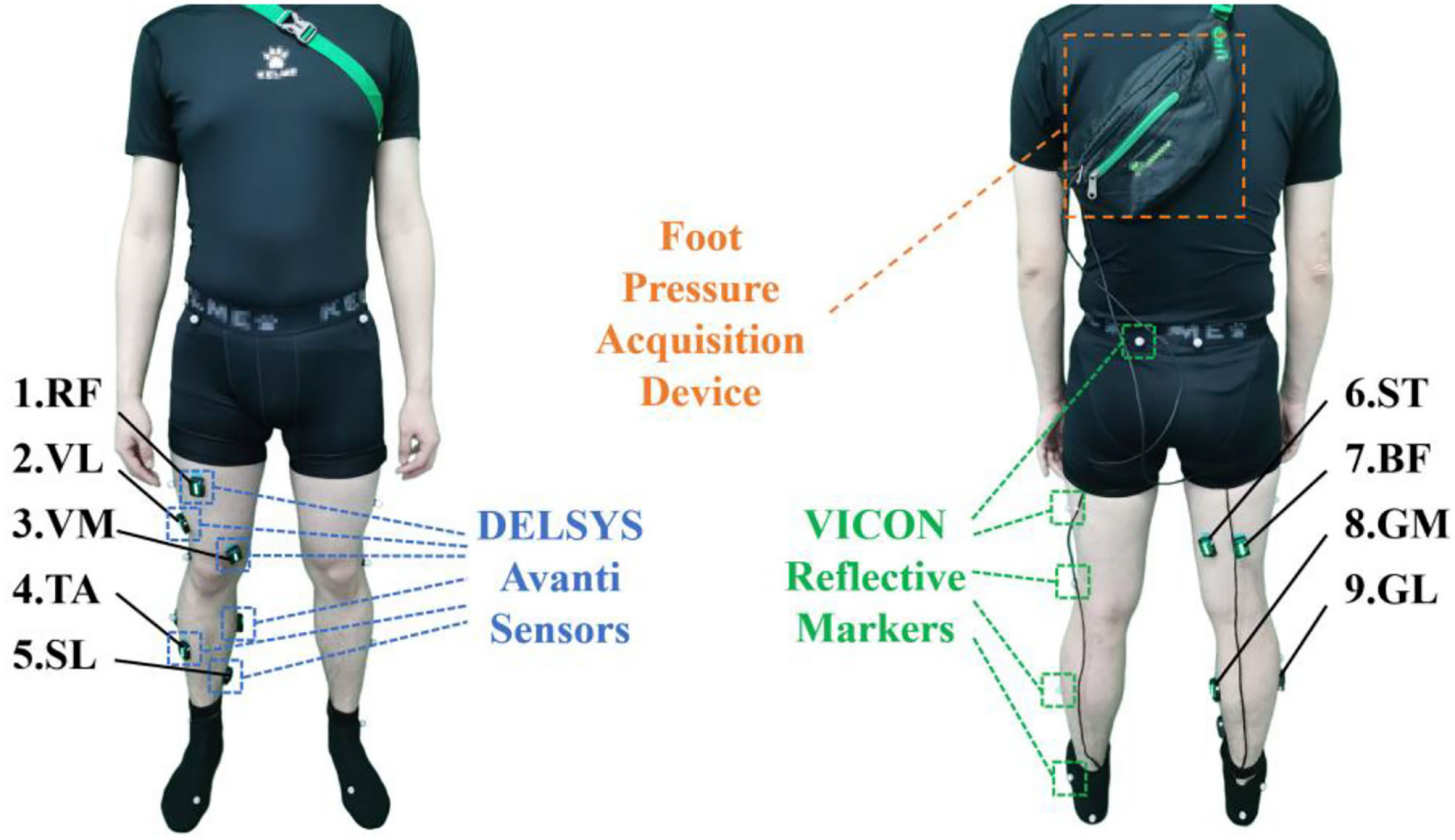
Sensor attachment of the subjects.

In this study, we constructed two datasets for the experimental protocol. In the first dataset, ten subjects were involved to performed the level-walking on a treadmill with a constant walking speed of 4.5 km/h. As shown in Figure 2, nine quadrupolar EMG electrodes were mounted on the lower-limb muscles, some of which have proved the validity in lower-limb motion decoding, corresponding to rectus femoris (RF), vastus lateralis (VL), vastus medialis (VM), tibialis anterior (TA), soleus (SL), biceps femoris (BF), semitendinosus (ST), gastrocnemius medial head (GM) and gastrocnemius lateral head (GL), with a sampling frequency of 1111.11 Hz. In order to decode the lower-limb kinematics of the subjects, 16 reflective markers were attached to the lower-limb, following the experimental scheme of the VICON user guide, and the lower-limb joint angles were collected with a sampling frequency of 100 Hz. In addition, two FSR sensors were attached to the heel and first metatarsal bone of the subject for phase labeling, foot pressure signals were collected with a sampling rate of 500 Hz.

In the second dataset, four of the ten subjects were recruited to participate in the experiments. With an ankle exoskeleton, the subjects performed the level-walking on a treadmill with a speed of 4.5 km/h. Based on the proposed muscle selection scheme described in Ankle Exoskeleton Frame section, a subset was selected from nine muscles to collect the EMG signals. The attachment of VICON markers and FSR sensors are the same as the first dataset. In both datasets, each subject was instructed to complete at least two trials of level-walking. Each trial lasted for 8 mins, and 15-min rest followed with each trial to avoid muscle fatigue.

### Ankle Exoskeleton Frame

**Figure 3:**
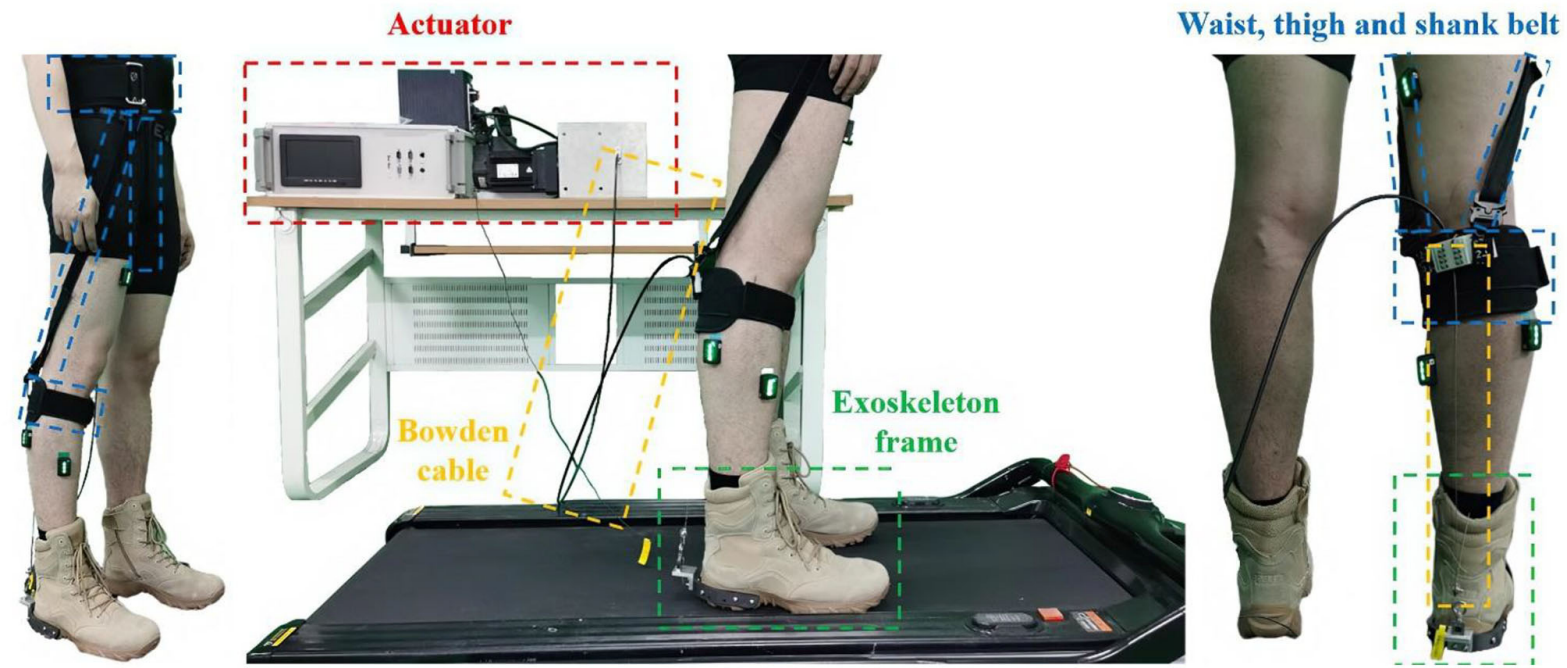
The exoskeleton frame implemented in the second dataset.

In this study, an ankle exoskeleton was implemented, which was shown in Figure 3. The designed ankle exoskeleton comprised a waist textile belt, two thigh textile belts, a shank textile belt, and an ankle end-effector mounted on the boot. The exoskeleton was actuated by a powerful motor, with the mechanical power transmitted through a flexible Boden cable tether which terminated at the heel.

The electronic control strategy of the exoskeleton was compiled in LabVIEW software and deployed to the Sbrio-9636 controller through a shared local area network, which was a single task mode control. At the event of heel-off, the motor pulled up on the end-effector through the Borden cable to provides an upward force of 100 N beneath the subject's heel, which assisted in reducing the plantarflexion forces provided by the subjects.

### Muscle Subset Selection

**Figure 4:**
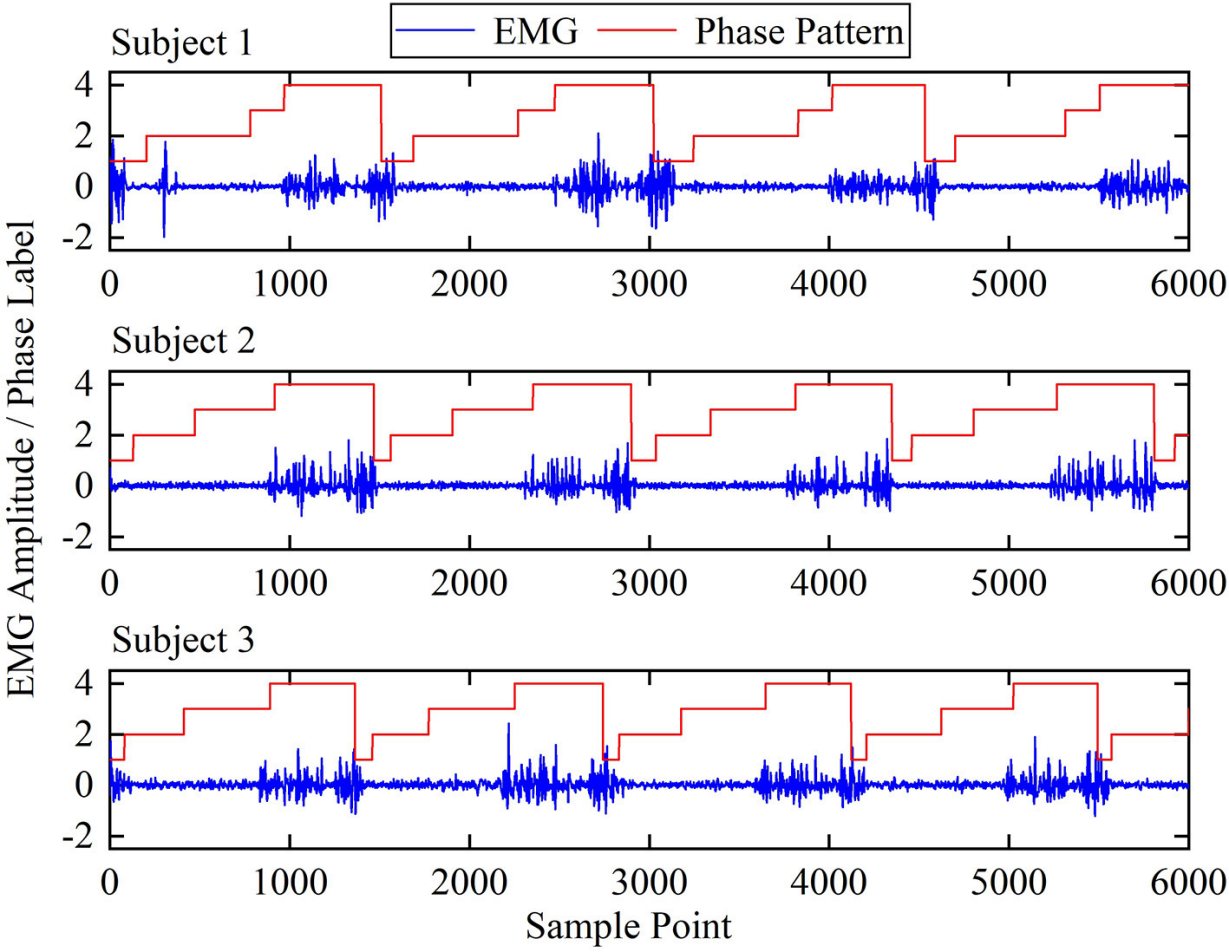
The EMG amplitude of tibialis anterior among different subjects.

sEMG signals are generated by nerve signals stimulating muscle activation, which contain massive human motion information. The amplitude and pulse duration of sEMG is highly correlated with the extent and duration of muscle activation, which varies in different phases. Figure 4 shows the sEMG amplitude of the tibialis anterior from different subjects, which was magnified 10,000 times. From the figure, a phenomenon can be found that the tibialis anterior is mainly activated in the fourth phase among the three subjects, which means that the muscles may not play a role in walking all the time. Instead, they activate at a certain time of the gait cycle. Moreover, although the sEMG amplitude and vibration frequency are different among the subjects, the timings of sEMG pulses in a gait cycle are roughly the same, which means different subjects may share a similar pattern of muscle activation (Chvatal et al., 2011).

Based on the assumption, a muscle-activation-based muscle selection scheme was proposed, which evaluated the effects of the muscles on phase recognition. Firstly, a standard manipulation was implemented to remove the motion artifact and other interference (Ngeo et al., 2014). Then, the signals were processed by full-wave rectification and normalized by dividing by the peak rectified EMG. A low-pass filter was carried out for the processed signal, as the frequency of muscle activation was much lower than that of EMG signals (Ding et al., 2011).

After the above manipulation, the neural activation u(i) of the ith processed EMG sample e(i) with TE sampling interval was calculated as follows:

(1) $$\begin{array}{r} \text{u}\left(\text{i}\right)=\alpha \times e\left(i-\frac{d}{T_{E}}\right)-\beta_{1}\times u\left(i-1\right){\;-\;\beta }_{2}\times u\left(i-1\right) \end{array}$$

where α, β_1_ and β_2_ are the recursive coefficients that maintain the stability of u(i), d is the time delay. Based on the neural activation derived from sEMG signal, the corresponding muscle activation a(t) was calculated by a simplified model (Lloyd and Besier, 2003). In equation (2), A is the nonlinear shape factor that varies between −3 and 0, with −3 represents highly exponential and 0 represents a linear relationship. This factor and the recursive coefficients can be determined by minimizing a mean-square error cost function (Ngeo et al., 2014). In this study, A is equal to −2.

(2) $$\begin{array}{r} \text{a}\left(\text{t}\right)=\frac{e^{\text{Au}\left(\text{t}\right)}-1}{{\text{e}}^{\text{A}}-1} \end{array}$$

Muscle activation sequence was calculated from EMG signals of each channel. Then, data of a gait cycle was extracted and segmented by different phases. After that, the average area Ai of muscle activation a(t) in phase i was calculated by:

(3) $$\begin{array}{r} A_{i}=\int_{t_{p}}^{T_{p}}a\left(t\right)dt\;i=1,2,3,4 \end{array}$$

Through the above calculation, A_i_ corresponding to four phases was obtained. In order to compare the activations of muscles in different phase more intuitively, a normalization operation was implemented to obtain the effect E_i_ of muscle to the ith phase. We would then evaluate the muscles based on the muscle effects, following the rule that at least four muscles should be selected, which have the highest activation in the corresponding four phases, and the muscles with similar activations in at least three phases would be discarded.

(4) $$\begin{array}{r} {\text{E}}_{\text{i}}\text{=}\frac{{\text{A}}_{\text{i}}}{\text{max(A)}}\;\text{i}=1,2,3,4 \end{array}$$

### Data Processing

After the optimal muscle subset was determined, the multi-channel EMG and joint angle streams were segmented by a continuous sliding window scheme, with a window length of 180 ms and a window increment of 40 ms (Englehart and Hudgins, 2003). In order to facilitate the phase classification and consider the time efficiency, the following time-domain features were extracted from each EMG segment, which were mean absolute value (MAV), zero crossing (ZC), slope signal change (SSC), and waveform length (WL). The effectiveness and the real-time capability of these features had already been verified in the related studies (He et al., 2011; Zhang et al., 2020). The feature vector x of a sliding window with the dimension of 4n is presented in the form of equation (5), where n represents the number of muscles and f denotes the extracted features from each muscle.

(5) $$\begin{array}{r} \text{x =}\left[{\text{f}}_{\text{1}},\;{\text{f}}_{\text{2}}{,\cdots \;,f}_{\text{n}}\right] \end{array}$$

### Phase Classifier and Angle Predictor

**Figure 5:**
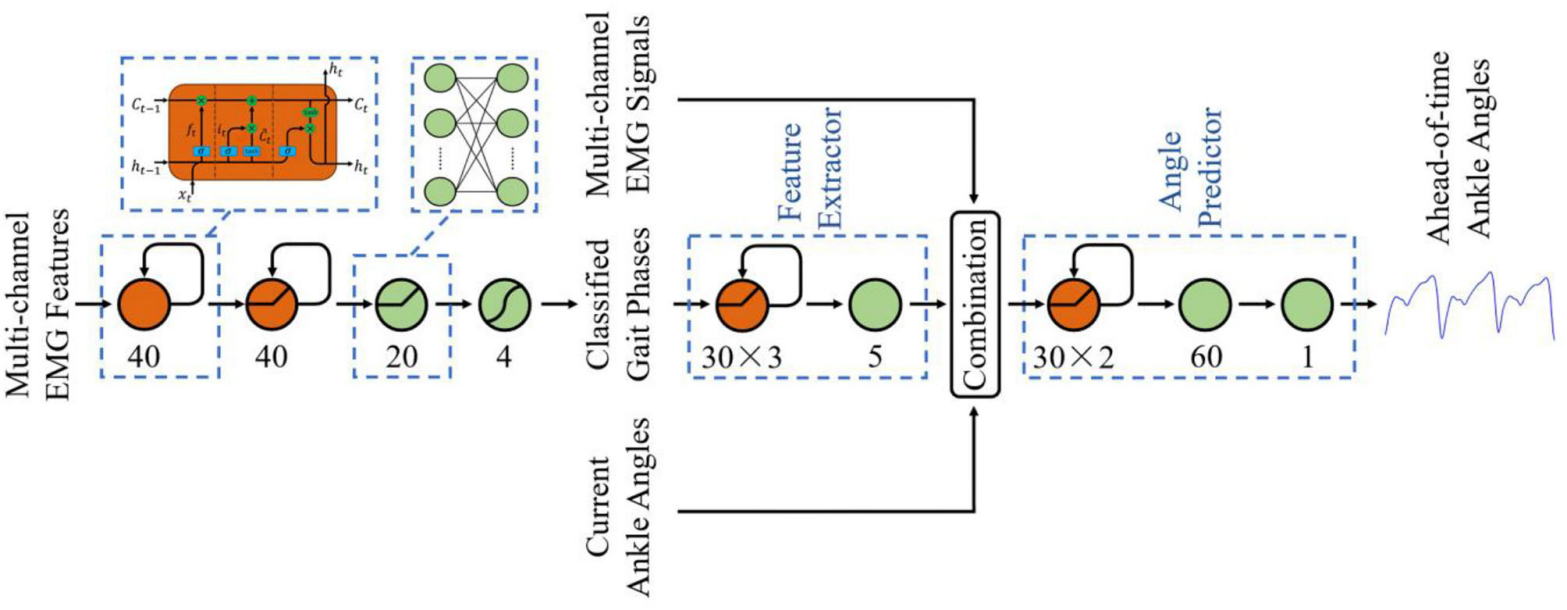
The structure of the phase classifier and ankle angle predictor.

For the classification of gait phases, options abound of machine learning, such as HMM (Evans and Arvind, 2014), LDA (Joshi et al., 2013), SVM (Ryu and Kim, 2014), etc. However, despite the verified effectiveness of these classifiers, they did not utilize the previous context of the gait phase, which was also an important element because of the quasi-periodicity of the changing phase state. Thus, we designed an LSTM-based phase classifier. The structure of the classifier was shown in Figure 5, consisting of an input layer with the dimension equal to the input features, two LSTM hidden layers of 40, a fully connected layer of 20, and a softmax layer of four corresponding to the gait phases. ReLU activation function was used to connect the LSTM layer, the fully connected layer, and the output layer. In order to prevent model overfitting, dropout regularization was applied after every fully connected hidden layer with probabilities of 0.5.

For the ahead-of-time prediction of ankle angles, LSTM was also implemented to leverage the quasi-periodicity of the changing ankle angles and gait phases. Different from the studies of phase classification, few feature extraction methods have been verified to be efficient for angle regression. Thus, as shown in Figure 5, a deep structure was designed, which combined a four-layer LSTM-based feature extractor (30-30-30-5) and a three-layer LSTM-based angle predictor (30-30-60-1). ReLU activation function was also implemented to connect the LSTM layer and the fully connected layer.

The models were tested on Nvidia Xavier Module Interface, with the overall running time for a time window was <30 ms. As the window increment was 40 ms, the prediction time of the angle predictor was set to 40 ms to compensate for the time delay and match the kinematics with the next incoming data stream.

### Evaluation Metrics

Several quantitative metrics were used to evaluate the performance of our method. The motion-decoding method we proposed is subject-specific. Thus, to improve the reliability of classification results while avoiding the problem of cross-subject, a modified leave-one-out cross-validation was carried out. Each time, one trial from a subject (defined in Data Acquisition and Experimental Protocol section) was regarded as the testing data, and the other trial from the same subject with all trials from other subjects were regarded as the training data. The procedure continued until each trial from each subject was tested. For all the evaluation processes, one-way ANOVA was implemented to validate the significant effect of a single variable on the results.

In order to verify the performance of the proposed classifier, the SVM classifier with the radial basis function kernel and the LDA classifier with the singular value decomposition solver were compared, which were implemented from the scikit-learn library. The feasibility of these classifiers have already been proved in the related research (He et al., 2011; Naik et al., 2018). In addition, classification accuracy (ACC) was used for the visualization of the performance.

SVR has been implemented to estimate the simultaneous DOFs of the joints in the related study, and outperformed ANN in myoelectric control tasks (Ameri et al., 2014). Thus, to verify the effectiveness of the proposed method, SVR was also implemented for angle prediction tasks in this study. The output of the predictor was a continuous time series of joint angles. Thus, the Pearson correlation coefficient (R-value) was implemented to quantify the linear relationship between the predicted and reference ankle angles:

(6) $$\begin{array}{r} \text{R =}\frac{\text{cov(}\theta_{\text{pre}},\;\theta_{\text{ref}}\text{)}}{\sigma_{\text{pre}}\sigma_{\text{ref}}} \end{array}$$

where θ_*pre*_ and θ_*ref*_ are defined as the predicted knee angles and reference knee angles, respectively. σ is the standard deviation, and *cov* represents the covariance. In addition to the similarity evaluation of the signals, the deviation and residual variance between the predicted and reference angles were estimated by the root mean square error (RMSE) and the normalized RMSE (NRMSE), where n denotes the total number of sampled data, and θ of equation (8) represents the predicted knee angles.

(7) $$\begin{array}{r} \text{RMSE =}\sqrt{\;\frac{1}{\text{n}}\sum {\left(\theta_{\text{pre}}-\theta_{\text{ref}}\right)}^{2}} \end{array}$$

(8) $$\begin{array}{r} \text{NRMSE =}\frac{\text{RMSE}}{\theta_{\text{max}}-\;\theta_{\text{min}}} \end{array}$$

## Results

To begin with, the effect of each muscle described in Ankle Exoskeleton Frame section was calculated, which was shown in Table 1. In order to avoid the error caused by abnormal phases, the whole procedure was repeated three times, and the corresponding E_i_ were averaged to obtain the result. Based on the muscle selection scheme, RF, TA, ST, GM, and GL were selected since they contained the muscles with the highest activation level in different phases, and each of them also had a discriminative activation level in another phase (shown in bold values), which might be beneficial for the phase classification task.

**Table 1:** Effects of nine muscles on different phase patterns.

**Muscle**	**Muscle effects on different gait phases**			
	**IC**	**FF**	**HO**	**TO**
**RF**	**1**	**0.49**	0.25	0.29
VM	1	0.35	0.27	0.33
VL	1	0.54	0.56	0.53
**TA**	**0.81**	0.18	0.21	**1**
SL	0.21	0.94	1	0.21
**ST**	**0.74**	0.24	0.23	**1**
BF	1	0.88	0.96	0.87
**GM**	0.16	**0.61**	**1**	0.16
**GL**	**0.44**	**0.78**	**1**	**0.18**

**Figure 6:**
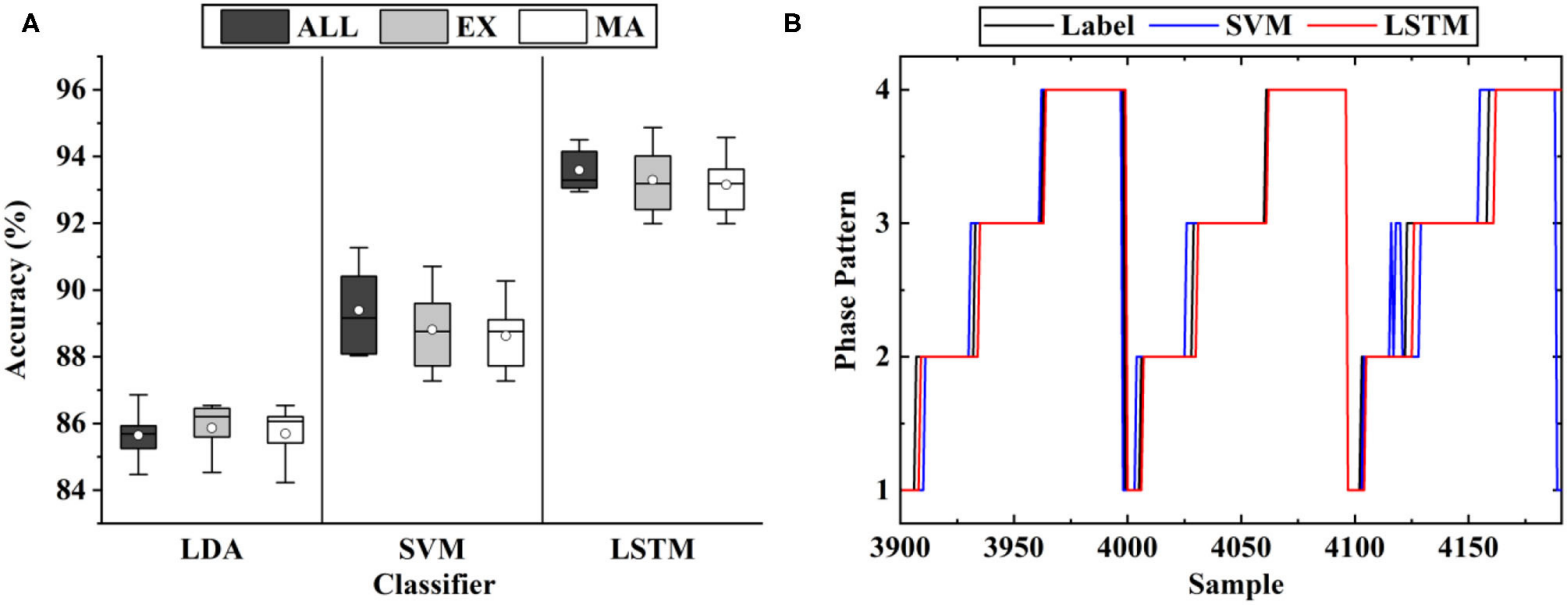
The classification results of gait phases: **(A)** The results of the different muscle sets, where MA represents the proposed muscle selection scheme, EX represents the exhaustive method and ALL represents all the nine muscles; **(B)** The representative results of the three classifiers.

Based on the selected muscles, the phase classification accuracy is shown in Figure 6, where MA represents the proposed muscle selection scheme. In order to verify the validity of the proposed method, the exhaustive method (EX) was compared. This method searched for optimal muscle subsets based on the classification accuracy, which was a time-consuming way. The result of nine muscles (ALL) was also presented to quantify the loss of information caused by muscle selection. In the figure, the average accuracy of MA (93.15% of LSTM) was a little lower than that of nine muscles (93.59% of LSTM), which meant that the excluded muscles contained some effective information, but no statistically significant difference was found (*P* > 0.05). In addition, the accuracy of MA was almost the same as that of EX (93.29% of LSTM, *P* > 0.05). When comparing the muscle subsets obtained by MA and EX, we discovered that the muscle subsets of six subjects were the same, while those of the other four were a little different, largely due to the error caused by muscle palpation and sensor location.

As shown in Figure 6B, the error mostly occurred in the transition of the phases, largely due to the ambiguity of the phase boundaries. As shown in the figure, the average classification accuracy of LSTM (93.15%) was significantly higher than that of SVM (88.63%) and LDA (85.69%). The same inference was also given when comparing the phase boundaries deduced by the classifiers. Thus, LSTM was implemented as the classifier in the following experiments.

**Figure 7:**
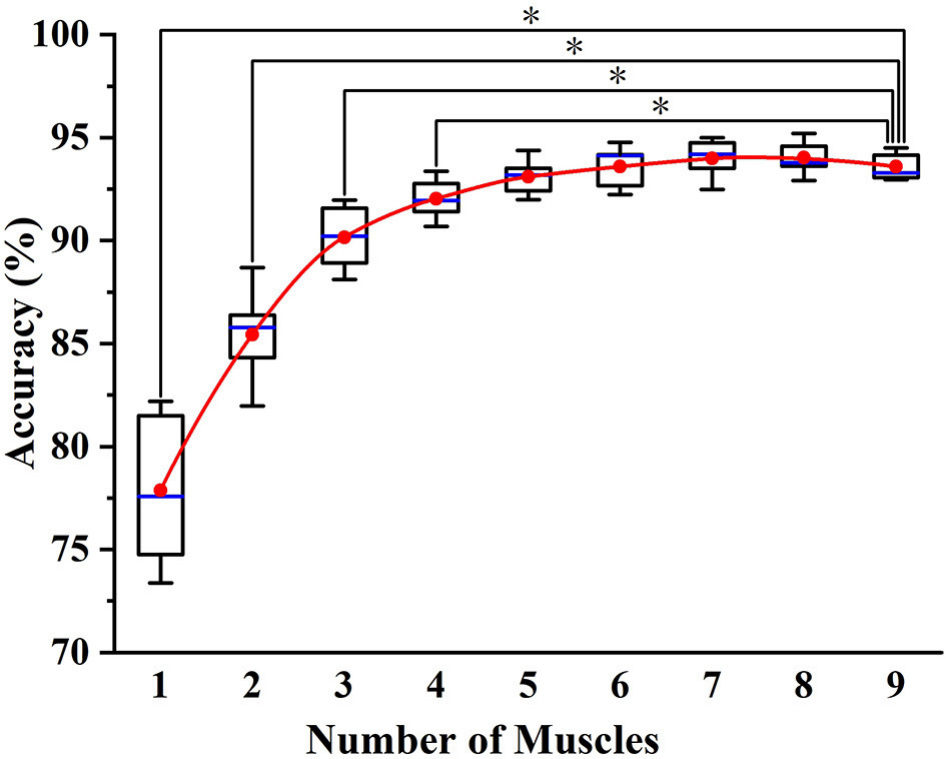
The classification results of the optimal muscle subsets based on different muscle numbers. * indicates a statistically significant difference (one-way ANOVA, *P* < 0.05).

Figure 7 shows the classification results of the optimal muscle subsets based on different muscle numbers. The result of five muscles was based on the muscle selection scheme, while the results of other muscle numbers were based on the exhaustive method. From the figure, a phenomenon could be found that the average accuracy of nine muscles (93.59%) was lower than that of eight muscles (94.02%) and seven muscles (93.98%), which was largely due to the muscle redundancy. In addition, the result of five muscles (93.15%) was not significantly different from that of nine muscles (P > 0.05), while that of four muscles was the opposite (*P* < 0.05). It meant that the selected muscle subset contained the minimum number of muscles while retaining the classification accuracy as much as possible.

**Figure 8:**
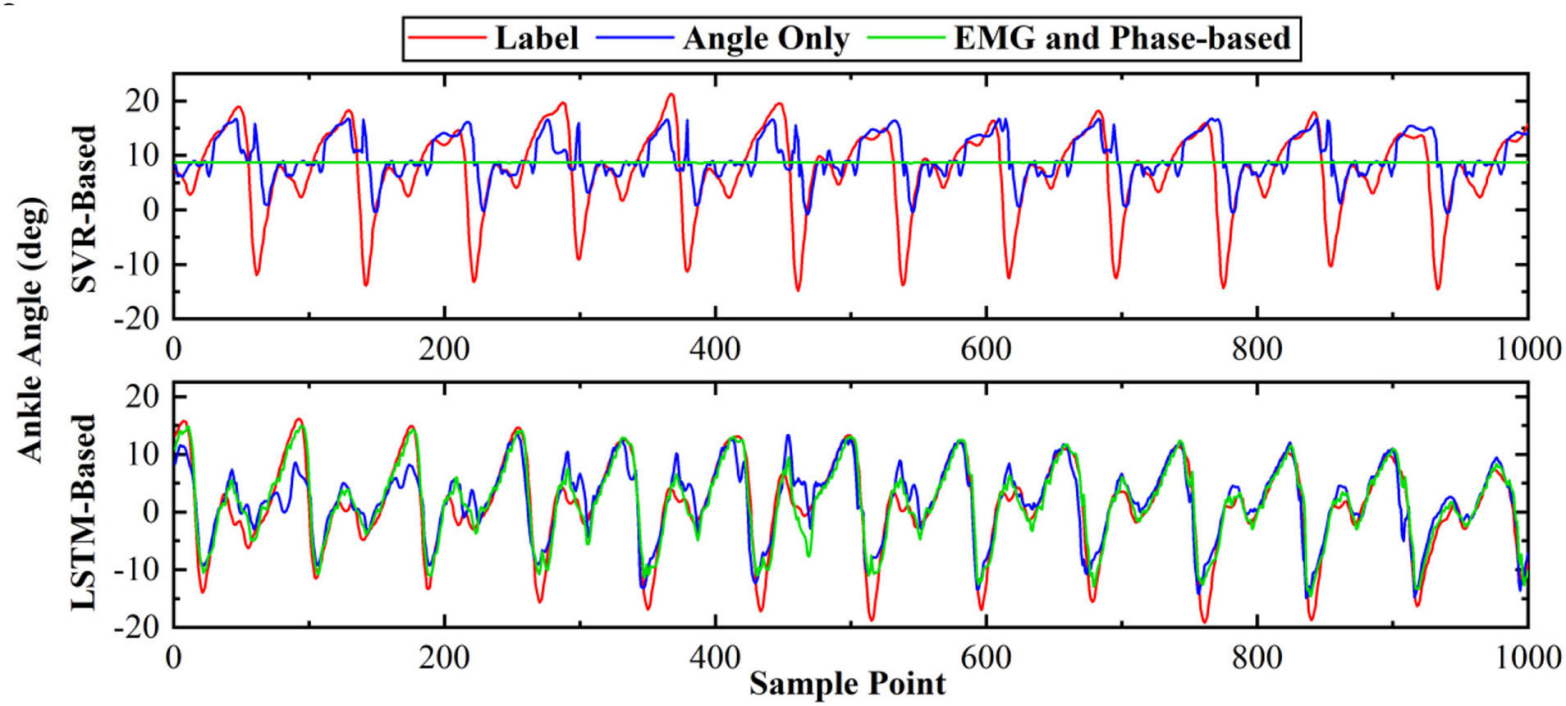
The representative results of different ankle angle predictors.

Figure 8 depicts the representative results of the angle predictor based on different data inputs. In the figure, Angle Only represent the inputs of one-channel current angles, while EMG and Phase-based represents those of five-channel sEMG, one-channel phases and one-channel current angles. As SVR is not able to extract features from sEMG, the feature set of **Muscle Subset Selection** section was implemented. As shown in the figure, the proposed LSTM-based predictor outperformed SVR in both Angle Only and EMG and Phase-based conditions.

**Figure 9:**
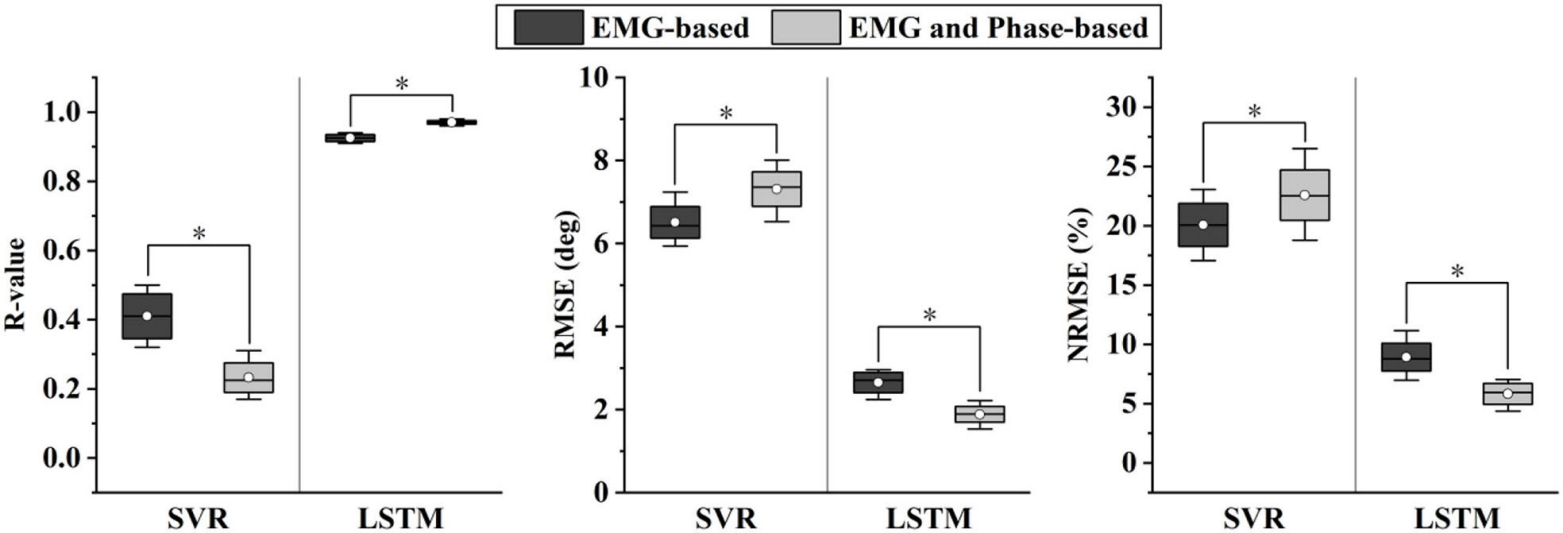
Comparison of different angle predictors based on two evaluation metrics. * indicates a statistically significant difference (one-way ANOVA, *P* < 0.05).

In Figure 9, the results of different data inputs were evaluated by the three metrics, where EMG-based represents the inputs of five-channel sEMG and one-channel current angles. As shown in the figure, the predicted angles of LSTM were significantly better than those of SVR (RMSE, 1.89° versus 6.51°; NRMSE, 20.07 versus 5.83%; *R*-value, 0.97 versus 0.41). For LSTM, it is shown that the results of EMG and Phase-based outperformed those of EMG-based, and a significant difference was found in the comparison of the results (*P* < 0.05). Thus, the data stream of sEMG and phases, and LSTM-based predictor were implemented in the following experiments.

**Figure 10:**
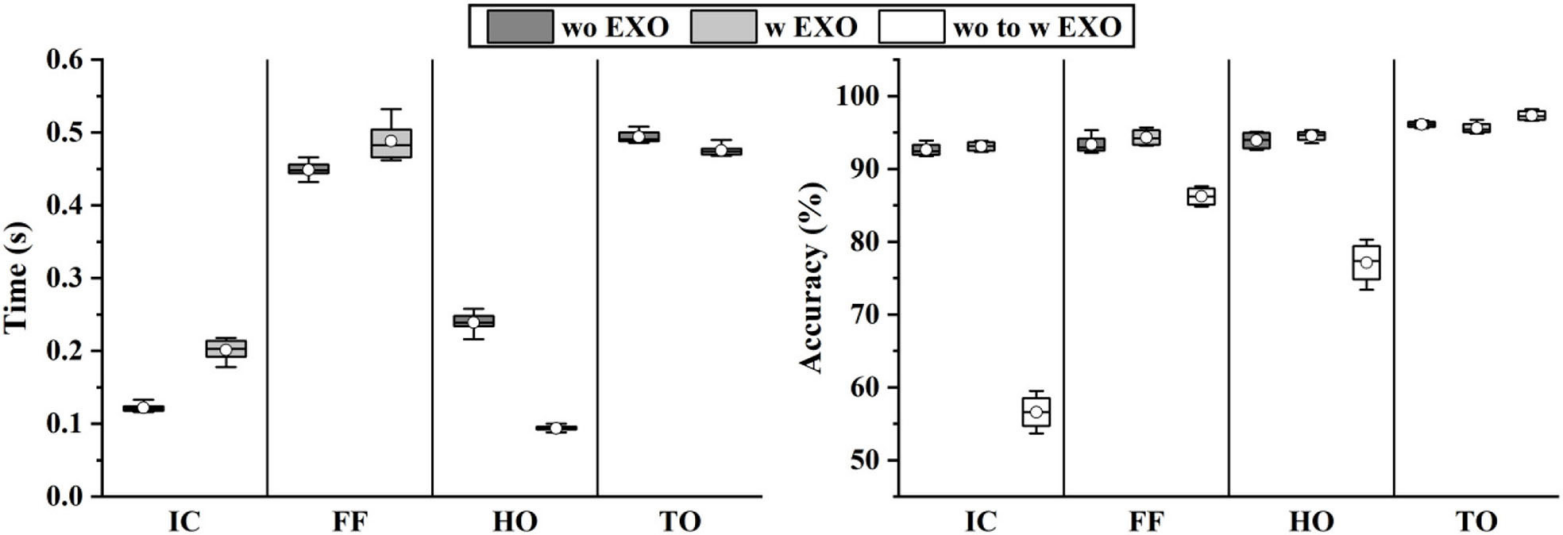
The duration and phase classification results involving the exoskeleton.

The effects of exoskeletons on phases have been quantified in Figure 10, where wo to w Exo represents that the model was trained in wo Exo (without exoskeleton) condition and tested in w Exo (with exoskeleton) condition. When the classifier was trained and tested in a single condition, the accuracy is quite high and stable, exhibiting that the muscle recruitment pattern of w Exo is as stationary as that of wo Exo. However, when the training and testing sets came from different conditions, the accuracy declined significantly. The most influenced phases were the IC (92.63–56.61%) and HO (93.91–77.12%), which corresponded to the difference in phase duration. A possible reason for this significant decline is that the altered muscle function significantly affects the distribution of sEMG, which have been reported in the related studies (Sylos-Labini et al., 2014; Li et al., 2019).

**Figure 11:**
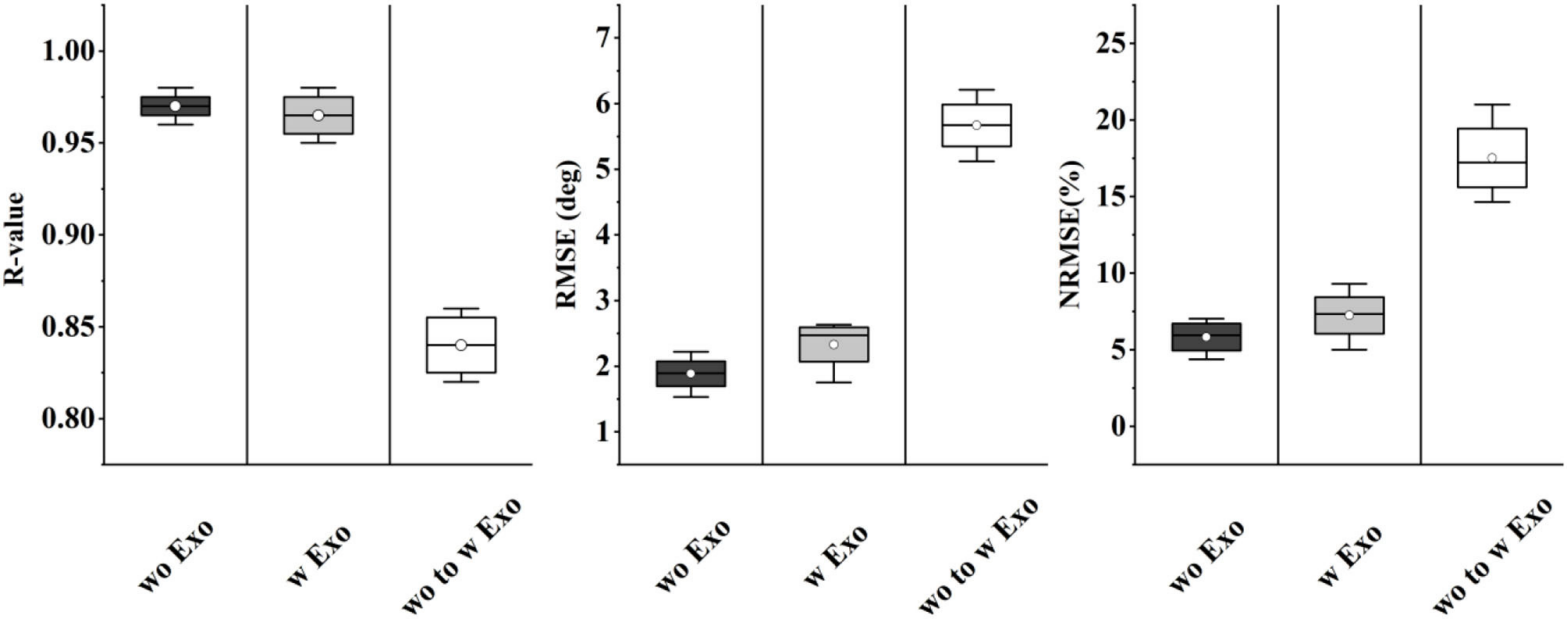
The prediction results involving the exoskeleton.

As shown in Figure 11, the results of angle prediction also supported the above view. In order to control the number of variables, the input phases of the predictor were the labels. Similar to the phase classifier, the angle predictor performed quite well in the single condition, but the accuracy declined significantly when the training and testing set came from different conditions (RMSE, 1.89°-5.68°; NRMSE, 5.83–17.52%; *R*-value, 0.97–0.84).

**Figure 12:**
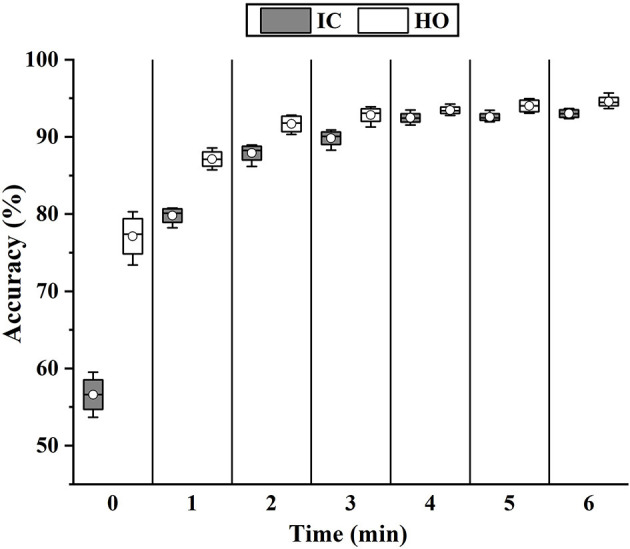
The change of classification accuracy when more data of w Exo was added to update the fine-tuning-based classifier.

In order to investigate the difference in muscle function in the two conditions and pursue a potential solution to the decline of accuracy, we adopted the fine-tuning method to update the classifier. Each time, 1 min w Exo data was added to update the model, which had already been trained by wo Exo data. The rest of the w Exo data was regarded as the testing set. As shown in Figure 12, the accuracies of phase IC and HO significantly increased (IC, 55.61–79.81%; HO, 77.12–87.13%) when the model was updated by 1-min data. In addition, the accuracy gradually stabilized when 4-min data was added, and the accuracy was roughly the same as that in the single w Exo condition (IC, 92.87 versus 92.63%; FF, 93.45 versus 93.91%).

**Figure 13:**
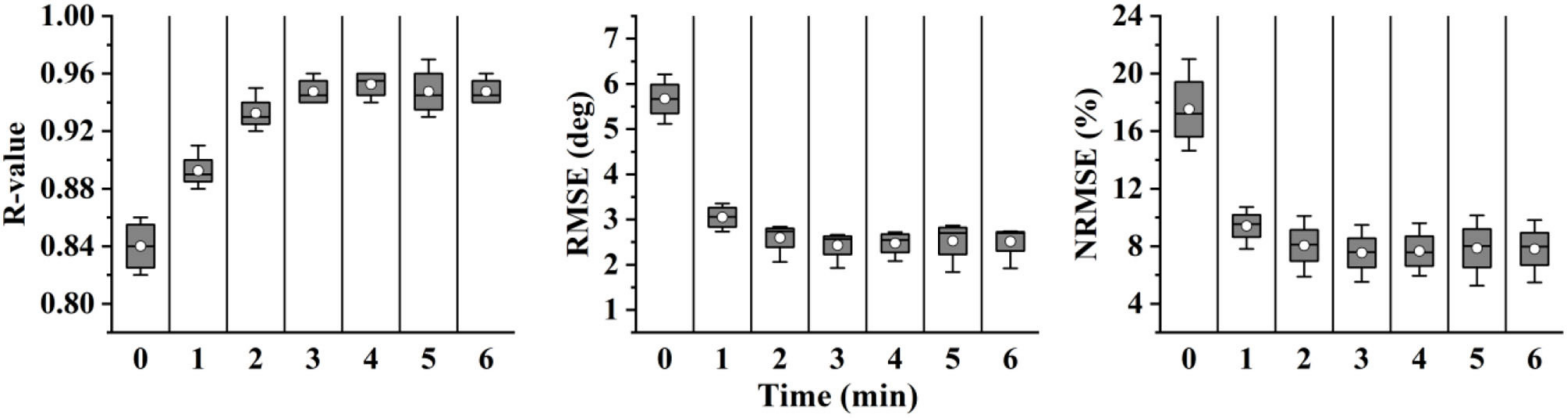
The change of prediction performance when more data of w Exo was added to update the fine-tuning-based predictor.

The results of fine-tuning-based angle prediction are shown in Figure 13. The accuracy was also significantly increased when 1-min data was added (RMSE, 5.68°-3.05°; NRMSE, 17.41–9.33%; *R*-value, 0.84–0.90), and gradually stabilized when 2-min data was added. Although the performance was not as good as that of only w Exo condition (RMSE, 2.52° versus 1.89°; NRMSE, 7.51 versus 5.83%; *R*-value, 0.95 versus 0.97), it was accurate enough to perform the ahead-of-time angle prediction.

## Discussion

As noted in the study, we proposed a novel sEMG and phase-based angle predictor and compared the contributions akin to ours. Through the muscle selection scheme, we reduced the number of muscles from nine to five, and the changes have little effect on the accuracy. The proposed method, which combined phase recognition and ankle angle prediction, significantly outperformed the related methods. In addition, through the fine-tuning scheme, the feasibility of the method was also verified in the exoskeleton condition, which effectively counteracted the signal distribution changes caused by exoskeleton assistance.

For data dimension reduction tasks, related studies either directly projected the data to the lower-dimensional space or selected the features that would best discriminate various movements via source estimates (Chu et al., 2007; Naik et al., 2018). Based on evaluating the muscle effects in each gait phase, we both reduced the dimension of data and the number of sensors. In addition, a surprising result is shown in Figure 7, exhibiting that the accuracy of nine muscles is slightly lower than that of eight and seven muscles. It indicated that some muscles might be not beneficial or even adverse to phase classification. In general, a viewpoint can be summarized that for phase classification, it is preferable to construct a muscle set with the activation of the muscles that are discriminative in different phases, rather than add as many muscles as possible to allow the classifiers to search for a complete muscle-phase relationship.

Various studies have been proposed for motion-decoding tasks, such as the discrete locomotion and gait phase recognition (Godiyal et al., 2018a,b), or continuous kinematic and dynamic estimation (Lloyd and Besier, 2003; Yi et al., 2018). However, the control of an exoskeleton can be enhanced if information in the future is available. Recently, Tanghe et al. proposed an IMU-based kinematics predictor, which was oriented to exoskeletons (Tanghe et al., 2020). Compared with their work, we leveraged the prior knowledge of the gait phase and EMD for kinematics prediction. In addition, transfer learning can be easily applied to the proposed data-driven method, especially when data distribution changes due to the intervention of exoskeletons. As shown in Figure 8, the results of sEMG and phase-based were significantly better than those of angle-based. The possible reason is twofold. Firstly, the EMD property of sEMG provides the ahead-of-time information for the prediction of the incoming ankle angles, which has been extracted by the deep LSTM-based feature extractor. Secondly, the joint training process both optimize the feature extractor and angle predictor, and reinforces the correlation between sEMG signals, phases, and ankle angles. In addition, the effect of phase priori for angle prediction was also tested. The results shown in Figure 9 suggested that besides sEMG, the gait phase also provided the prior knowledge for angle prediction, thus further improved the prediction accuracy.

In this study, we investigated and quantified how the exoskeleton affected the sEMG-based motion decoding methods. As shown in Figures 10, 11, the results significantly declined when the training and testing data came from different conditions. The interference of the exoskeleton was considered to be the main reason for this phenomenon. First, the exoskeleton disturbed the walking patterns, which was shown in Figure 10A and was also reported in Tanghe et al. (2020). Second, the exoskeleton altered the muscle recruitment patterns, exhibiting that the muscles were not restricted to the fixed synergistic patterns. They will selectively modulate the activity given the external interference, instead (Steele et al., 2017).

In order to seek a potential solution to this problem, the fine-tuning scheme was implemented to update the model. As shown in Figures 12, 13, when adding 1-min w Exo data into the training set, the performance of the phase classifier and angle predictor significantly increased. This phenomenon suggested that a correlation might exist between the altered muscle synergy and the original one, thus enabling the fine-tuning of the model with a small size of data. In addition, the performance of the models that were updated through 4-min data was close to that of the models based on whole data of the trials with the exoskeleton, which validated the feasibility of the proposed scheme.

Despite the LSTM-based angle predictor achieved good performance in ahead-of-time ankle angle prediction, there is still room to improve the validity of the method. Since the phases were inputs of the angle predictor, the error caused by phase misclassification would affect the performance of angle prediction. Therefore, the proper post-processing procedure is beneficial to reduce the occurrence of the accumulated error. In addition, even though the fine-tuning scheme was validated to be efficient for the accuracy decline of the model caused by exoskeleton interference, the need for data of trials with the exoskeleton is still inconvenient. The adaption of motion-decoding methods from normal walking to exoskeleton-involved walking would be an important pointer for future research, which necessitates a larger dataset with sufficient subjects and more investigation of the effects of the exoskeleton on muscle functions.

## Conclusion

In this study, we proposed a novel ankle angle predictor, which presented the prediction of kinematics. First of all, a reduced set of muscles was selected by the proposed muscle selection scheme, which was meant to reduce the data dimension in the muscle level. Secondly, An LSTM-based phase classifier was designed to assign the sEMG to four phases. Finally, with the aid of sEMG and phases, the proposed angle predictor presents the ahead-of-time prediction based on the measured ankle angles.

In order to investigate the perturbance of the exoskeleton to the proposed method, the method was trained on a dataset for normal walking and tested on a dataset for walking with an exoskeleton. From the result, we showed that the method is effective for both phase classification and angle prediction on the training set, while the accuracy significantly declined on the testing set. In order to compensate for the decline of accuracy, a fine-tuning scheme was implemented. After the model update manipulation, the accuracy of phase classification and angle prediction on the testing set had significantly increased and close to that on the training set.

The method enabled the quantitative compensation for the time delay of the exoskeletons, which offers opportunities to achieve a more accurate and smooth control system. In addition, the study enabled us to comprehend the inherent limitations for the applications of the motion-decoding method to the exoskeletons. Being cognizant of these factors, our future work objective is to explore the physiological mechanism of human-exoskeleton interaction and seek for a solution to allow the exoskeletons to adapt to a new subject without the pretraining procedure.

## Data Availability Statement

The raw data supporting the conclusions of this article will be made available by the authors, without undue reservation.

## Ethics Statement

The studies involving human participants were reviewed and approved by Chinese Ethics Committee of Registering Clinical Trials. The patients/participants provided their written informed consent to participate in this study.

## Author Contributions

BW and CY: conceptualization and writing–original draft preparation. BW and ZD: methodology. BW: software and visualization. BW and FJ: validation and investigation. BW and HG: formal analysis and data curation. FJ: resources, supervision, project administration, and funding acquisition. HG, ZW, and JZ: writing–review and editing. All authors have read and agreed to the published version of the manuscript.

## Conflict of Interest

The authors declare that the research was conducted in the absence of any commercial or financial relationships that could be construed as a potential conflict of interest.

## Publisher's Note

All claims expressed in this article are solely those of the authors and do not necessarily represent those of their affiliated organizations, or those of the publisher, the editors and the reviewers. Any product that may be evaluated in this article, or claim that may be made by its manufacturer, is not guaranteed or endorsed by the publisher.

## References

[B1] Ameri A. Kamavuako E. N. Scheme E. J. Englehart K. B. Parker P. A. (2014). Support vector regression for improved real-time, simultaneous myoelectric control. IEEE Trans. Neural Syst. Rehabil. Eng. 22, 1198–1209. 10.1109/TNSRE.2014.2323576 24846649

[B2] Awad L. N. Bae J. O'Donnell K. De Rossi S. M. M. Hendron K. Sloot L. H.. (2017). A soft robotic exosuit improves walking in patients after stroke. Sci. Transl. Med. 9:eaai9084. 10.1126/scitranslmed.aai9084 28747517

[B3] Bonato P. (2010). Wearable sensors and systems. IEEE Eng. Med. Biol. Mag. 29, 25–36. 10.1109/MEMB.2010.936554 20659855

[B4] Chadwell A. Diment L. Micó-Amigo M. Morgado Ramírez D. Z. Dickinson A. Granat M.. (2020). Technology for monitoring everyday prosthesis use: a systematic review. J. NeuroEng. Rehabil. 17:93. 10.1186/s12984-020-00711-4 32665020 PMC7362458

[B5] Chu J.-U. Moon I. Lee Y.-J. Kim S.-K. Mun M.-S. (2007). A supervised feature-projection-based real-time emg pattern recognition for multifunction myoelectric hand control. IEEE/ASME Trans. Mechatron. 12, 282–290. 10.1109/TMECH.2007.897262

[B6] Chvatal S. A. Torres-Oviedo G. Safavynia S. A. Ting L. H. (2011). Common muscle synergies for control of center of mass and force in nonstepping and stepping postural behaviors. J. Neurophysiol. 106, 999–1015. 10.1152/jn.00549.2010 21653725 PMC3154805

[B7] Ding Q. C. Xiong A. B. Zhao X. G. Han J. D. (2011). A novel EMG-driven state space model for the estimation of continuous joint movements, in IEEE, 2891–2897. 10.1109/ICSMC.2011.6084104

[B8] Englehart K. Hudgin B. Parker P. A. (2001). A wavelet-based continuous classification scheme for multifunction myoelectric control. IEEE Trans. Biomed. Eng. 48, 302–311. 10.1109/10.914793 11327498

[B9] Englehart K. Hudgins B. (2003). A robust, real-time control scheme for multifunction myoelectric control. IEEE Trans. Biomed. Eng 50, 848–854. 10.1109/TBME.2003.813539 12848352

[B10] Evans R. L. Arvind D. K. (2014). Detection of gait phases using orient specks for mobile clinical gait analysis, in 2014 11th International Conference on Wearable and Implantable Body Sensor Networks (Zuirch, Switzerland: IEEE), 149–154.

[B11] Fan Y. Yin Y. (2009). Mechanism design and motion control of a parallel ankle joint for rehabilitation robotic exoskeleton, in 2009 IEEE International Conference on Robotics and Biomimetics (ROBIO) (Guilin, China: IEEE), 2527–2532.

[B12] Ferris D. P. Sawicki G. S. Daley M. A. (2007). A physiologist's perspective on robotic exoskeletons for human locomotion. Int. J. Human. Robot. 04, 507–528. 10.1142/S0219843607001138 PMC2185037 18185840

[B13] Godiyal A. K. Mondal M. Joshi S. D. Joshi D. (2018a). Force myography based novel strategy for locomotion classification. IEEE Trans. Human-Mach. Syst. 48, 648–657. 10.1109/THMS.2018.2860598

[B14] Godiyal A. K. Verma H. K. Khanna N. Joshi D. (2018b). A force myography-based system for gait event detection in overground and ramp walking. IEEE Trans. Instrum. Meas. 67, 2314–2323. 10.1109/TIM.2018.2816799

[B15] He Huang Fan Zhang Hargrove L. J. Zhi Dou Rogers D. R. Englehart K. B. (2011). Continuous locomotion-mode identification for prosthetic legs based on neuromuscular–mechanical fusion. IEEE Trans. Biomed. Eng. 58, 2867–2875. 10.1109/TBME.2011.2161671 21768042 PMC3235670

[B16] Hioki M. Kawasaki H. (2012). Estimation of finger joint angles from sEMG using a neural network including time delay factor and recurrent structure. ISRN Rehabi. 2012, 1–13. 10.5402/2012/604314

[B17] Hudgins B. Parker P. Scott R. N. (1993). A new strategy for multifunction myoelectric control. IEEE Trans. Biomed. Eng. 40, 82–94. 10.1109/10.204774 8468080

[B18] Joshi C. D. Lahiri U. Thakor N. V. (2013). Classification of gait phases from lower limb EMG: application to exoskeleton orthosis, in 2013 IEEE Point-of-Care Healthcare Technologies (PHT) (Bangalore, India: IEEE), 228–231. 10.1109/PHT.2013.6461326

[B19] Kazerooni H. (2008). Exoskeletons for human performance augmentation, in Springer Handbook of Robotics, eds Siciliano B. Khatib O. (Berlin, Heidelberg: Springer), 773–793. 10.1007/978-3-540-30301-5_34

[B20] Kim J. Lee G. Heimgartner R. Arumukhom Revi D. Karavas N. Nathanson D.. (2019). Reducing the metabolic rate of walking and running with a versatile, portable exosuit. Science 365, 668–672. 10.1126/science.aav7536 31416958

[B21] LeCun Y. Bengio Y. Hinton G. (2015). Deep learning. Nature 521, 436–444. 10.1038/nature14539 26017442

[B22] Li Z. Liu H. Yin Z. Chen K. (2019). Muscle synergy alteration of human during walking with lower limb exoskeleton. Front. Neurosci. 12:1050. 10.3389/fnins.2018.01050 30760972 PMC6361853

[B23] Lloyd D. G. Besier T. F. (2003). An EMG-driven musculoskeletal model to estimate muscle forces and knee joint moments in vivo. J. Biomech. 36, 765–776. 10.1016/S0021-9290(03)00010-1 12742444

[B24] Moreno J. C. Barroso F. Farina D. Gizzi L. Santos C. Molinari M.. (2013). Effects of robotic guidance on the coordination of locomotion. J. NeuroEng. Rehabil. 10:79. 10.1186/1743-0003-10-79 23870328 PMC3724716

[B25] Naik G. R. Selvan S. E. Arjunan S. P. Acharyya A. Kumar D. K. Ramanujam A.. (2018). An ICA-EBM-based sEMG classifier for recognizing lower limb movements in individuals with and without knee pathology. IEEE Trans. Neural Syst. Rehabil. Eng. 26, 675–686. 10.1109/TNSRE.2018.2796070 29522411

[B26] Ngeo J. G. Tamei T. Shibata T. (2014). Continuous and simultaneous estimation of finger kinematics using inputs from an EMG-to-muscle activation model. J. Neuroeng. Rehabil. 11:122. 10.1186/1743-0003-11-122 25123024 PMC4148535

[B27] Novak D. Riener R. (2015). A survey of sensor fusion methods in wearable robotics. Rob. Auton. Syst. 73, 155–170. 10.1016/j.robot.2014.08.012

[B28] Resnik L. Huang H. Winslow A. Crouch D. L. Zhang F. Wolk N. (2018). Evaluation of EMG pattern recognition for upper limb prosthesis control: a case study in comparison with direct myoelectric control. J. NeuroEng. Rehabil. 15:23. 10.1186/s12984-018-0361-3 29544501 PMC5856206

[B29] Ryu J. H. Kim D. H. (2014). Multiple gait phase recognition using boosted classifiers based on sEMG signal and classification matrix, in International Conference on Ubiquitous Information Management and Communication, 1–4.

[B30] Sankai Y. (2010). HAL: Hybrid Assistive Limb Based on Cybernics, in Robotics Research, eds Kaneko M. Nakamura Y. (Berlin, Heidelberg: Springer), 25–34. 10.1007/978-3-642-14743-2_3

[B31] Scheme E. Englehart K. (2011). Electromyogram pattern recognition for control of powered upper-limb prostheses: State of the art and challenges for clinical use. JRRD 48:643. 10.1682/JRRD.2010.09.0177 21938652

[B32] Seel T. Raisch J. Schauer T. (2014). IMU-based joint angle measurement for gait analysis. Sensors 14, 6891–6909. 10.3390/s140406891 24743160 PMC4029684

[B33] Steele K. M. Jackson R. W. Shuman B. R. Collins S. H. (2017). Muscle recruitment and coordination with an ankle exoskeleton. J. Biomech. 59, 50–58. 10.1016/j.jbiomech.2017.05.010 28623037 PMC5644499

[B34] Sylos-Labini F. La Scaleia V. d'Avella A. Pisotta I. Tamburella F. Scivoletto G.. (2014). EMG patterns during assisted walking in the exoskeleton. Front. Hum. Neurosci. 8, 1–12. 10.3389/fnhum.2014.00423 24982628 PMC4058900

[B35] Taborri J. Palermo E. Rossi S. Cappa P. (2016). Gait partitioning methods: a systematic review. Sensors 16:66. 10.3390/s16010066 PMC4732099 26751449

[B36] Tanghe K. De Groote F. Lefeber D. De Schutter J. Aertbelien E. (2020). Gait trajectory and event prediction from state estimation for exoskeletons during gait. IEEE Trans. Neural Syst. Rehabil. Eng. 28, 211–220. 10.1109/TNSRE.2019.2950309 31675336

[B37] Viteckova S. Kutilek P. Jirina M. (2013). Wearable lower limb robotics: A review. Biocybern. Biomedi. Eng. 33, 96–105. 10.1016/j.bbe.2013.03.005

[B38] Vu H. Gomez F. Cherelle P. Lefeber D. Nowé A. Vanderborght B. (2018). ED-FNN: a new deep learning algorithm to detect percentage of the gait cycle for powered prostheses. Sensors 18:2389. 10.3390/s18072389 PMC6068484 30041421

[B39] Williams T. W. (1990). Practical methods for controlling powered upper-extremity prostheses. Assis. Technol. 2, 3–18. 10.1080/10400435.1990.10132142 10149040

[B40] Xia P. Hu J. Peng Y. (2018). EMG-based estimation of limb movement using deep learning with recurrent convolutional neural networks: EMG-based estimation of limb movement. Artif. Organs 42, E67–E77. 10.1111/aor.13004 29068076

[B41] Yan T. Cempini M. Oddo C. M. Vitiello N. (2015). Review of assistive strategies in powered lower-limb orthoses and exoskeletons. Rob. Auton. Syst. 64, 120–136. 10.1016/j.robot.2014.09.032

[B42] Yang X. Lihua G. Yang Z. Gu W. (2008). Lower extreme carrying exoskeleton robot adative control using wavelet neural networks, in IEEE, 399–403. 10.1109/ICNC.2008.754

[B43] Yi C. Jiang F. Zhang S. Guo H. Yang C. Ding Z.. (2021). Continuous prediction of lower-limb kinematics from multi-modal biomedical signals. IEEE Trans. Circuits Syst. Video Technol. 1–1. 10.1109/TCSVT.2021.3071461

[B44] Yi C. Ma J. Guo H. Han J. Gao H. Jiang F.. (2018). Estimating three-dimensional body orientation based on an improved complementary filter for human motion tracking. Sensors 18:3765. 10.3390/s18113765 PMC6263778 30400359

[B45] Zhang K. Wang J. de Silva C. W. Fu C. (2020). Unsupervised Cross-Subject Adaptation for Predicting Human Locomotion Intent. IEEE Trans. Neural Syst. Rehabil. Eng. 28, 646–657. 10.1109/TNSRE.2020.2966749 31944980

